# Different Strategies of Stabilization of Vanadium Oxidation States in Lagao_3_ Nanocrystals

**DOI:** 10.3389/fchem.2019.00520

**Published:** 2019-07-23

**Authors:** Karolina Kniec, Lukasz Marciniak

**Affiliations:** Institute of Low Temperature and Structure Research, Polish Academy of Sciences, Wroclaw, Poland

**Keywords:** vanadium, charge compensation, citric acid, luminescence, nanocrystals

## Abstract

The spectroscopic properties of LaGaO_3_, doped with V ions, were examined in terms of the possibility of the stabilization of particular vanadium oxidation states. It was shown that three different approaches may be applied in order to control the ionic charge of vanadium, namely, charge compensation, via incorporation of Mg^2+^/Ca^2+^ ions, citric acid (CA)-assisted synthesis, with various CA concentrations and grain size tuning through annealing temperature regulation. Each of utilized method enables the significant reduction of V^5+^ emission band at 520 nm associated with the V^4+^→ O^2−^ CT transition in respect to the ^2^E→ ^2^T_2_ emission band of V^4+^ at 645 nm and ^1^E_2_ → ^3^T_1g_ emission band of V^3+^ at 712 nm. The most efficient V oxidation state stabilization was obtained by the use of grain size modulation, which bases on fact of different localization of the V ions of given charge in the nanoparticles. Moreover, the CA-assisted synthesis of LaGaO_3_:V determines V valence states but also provides significant separation of the nanograins. It was found that superior charge compensation was achieved when Mg^2+^ ions were introduced in the matrix, due the more efficient lability, resulting from the comparable ionic radii between Mg^2+^ and V ions.

## Introduction

It is well-known that the electronic configuration of optically active ions and thus their spectroscopic properties depend strongly on the their oxidation state (Weber and Riseberg, 1971; Felice et al., 2001; Gupta et al., 2014; Matin et al., 2017; Drabik et al., 2018; Kniec and Marciniak, 2018a,b; Trejgis and Marciniak, 2018). These features may in turn be influenced by many factors, such as type of the lattice, in which the ions are embedded and coordination number of the substituted ion, synthesis method, size of phosphor grain etc. (McKittrick et al., 1999; Azkargorta et al., 2016; Marciniak et al., 2017; Kniec and Marciniak, 2018b; Zhang et al., 2018). The difference in the spectroscopic properties of optically active ions depending on the oxidation state in the case of lanthanides in especially well-manifested for europium ions, which may occur in two 2^+^ and 3^+^ oxidation states. Emission spectra of its trivalent ions consist of narrow lines attributed to intraconfigurational f-f electronic transitions whose spectral position is almost independent on the type of host material. On the other hand emission of Eu^2+^ is characterized by broad d-d emission band of maxima which can be tuned by the modification of the crystal field strength (Peng and Hong, 2007; Mao et al., 2009; Mao and Wang, 2010; Sato et al., 2014; Zhang et al., 2015). Similar differences can be found in the case of transition metal (TM) ions, where electronic transitions between d states are responsible for luminescence. In turn the luminescence of TM on different oxidation state is conditioned by presence of either octahedral or tetrahedral local site symmetries, where the optically active ion is substituted (Grinberg et al., 2017; Cao et al., 2018; Elzbieciak et al., 2018). This points to the importance of the appropriate choice of the local ion's environment and thus the host material to obtain efficient luminescence. The valence state of TM influences their output color, what is the most frequently encountered in case of manganese and chromium ions (Brik et al., 2011; Cao et al., 2016, 2018; Elzbieciak et al., 2018). Cr^3+^ ions may reveal red and NIR luminescence ascribed to the ^2^E_g_ → ^4^A_2g_ spin-forbidden and ^4^T_2g_ → ^4^A_2g_ spin-allowed transition and (Struve and Huber, 1985; Brik et al., 2016; Elzbieciak et al., 2018), whereas Cr^4+^ ions exhibit emission in NIR region (around 1,100 nm), which is attributed to the ^3^A_2_ → ^3^T_1_ transition (Devi et al., 1996). Different emission color is also observed for manganese ions, where Mn^2+^, Mn^3+^ and Mn^4+^ ions show blue (^4^A_2_ → ^4^T_1_), yellow-orange (^4^T_1g_ → ^6^A_1g_) or red (^5^T_2_ → ^5^E) luminescence, respectively (Trejgis and Marciniak, 2018). This phenomenon is also observed for titanium ions, where Ti^4+^ and Ti^3+^ possess blue and red emission color, which related to the O^2−^ → T_2_ (CT, λ_em_ ~ 450 nm) and ^2^E_g_ → ^2^T_2g_ (λ_em_ ~ 800 nm) transitions, respectively (Martínez-Martínez et al., 2005; Pathak and Mandal, 2011; Drabik et al., 2018). The possibility of charge modulation is not only interesting in terms of spectroscopic tunability but also to get rid of some valence states, being marked by toxic properties and reducing the potential biological deployment, which is very important in case of chromium and cobalt ions (Buzea et al., 2007; Chowdhury and Yanful, 2010; Wang et al., 2012; Scharf et al., 2014).

One of the least known transition metal ion, whose optical properties strongly depend on the oxidation state is vanadium. Several oxidation states of vanadium can be found like V^5+^, V^4+^, V^3+^, V^2+^ of the 3d^0^, 3d^1^, 3d^2^, 3d^3^ electronic configuration, respectively. Recently we presented the potential application of V ions emission for luminescent nanothermometry, where V ions were incorporated into inorganic hosts, such as YAG (Y_3_Al_5_O_12_) (Kniec and Marciniak, 2018b) and LaGaO_3_ (Kniec and Marciniak, 2018a). As it was shown its high susceptibility to the thermal changes enables to detect the local temperatures with satisfactory sensitivity (Kniec and Marciniak, 2018a,b). Depending on the host material different V oxidation states were found, namely V^5+^, V^3+^ and V^5+^, V^4+^, V^3+^, for YAG and LaGaO_3_ lattices, respectively. What is more, an immense impact of synthesis method and annealing temperature on crystalline size, dispersion factor of the particles, size distribution were presented (Kniec and Marciniak, 2018a,b), which in turn lead to the presence of different V valence states, characterized by distinct luminescent properties and susceptibility to changes of local ion environment. Due to good spectral separation of emission bands of particular oxidation states of vanadium ions the qualitatively verification of their presence can be done basing on the analysis of the luminescent properties of vanadium doped phosphor. The V^5+^, V^4+^, and V^3+^ luminescence is related with V^4+^ → O^2−^ CT transition (λ_em_ = 520 nm), broad band ^2^E → ^2^T_2_ d-d electronic transition (λ_em_ = 645 nm) and narrow line ^1^E_2_ → ^3^T_1g_ d-d electronic transition (λ_em_ = 712 nm), respectively (Ryba-Romanowski et al., 1999a; Kniec and Marciniak, 2018a,b).

In the course of our previous studies it was found that broad emission band of V^5+^ with the maxima at around 497 nm (V^4+^ → O^2−^ CT emission) predominates in the emission spectra (Kniec and Marciniak, 2018a,b). However, as it was already proved the ^2^E → ^2^T_2_ emission of V^4+^ ions is characterized by higher susceptibility to luminescence thermal quenching and hence reveals the best performance to non-contact readout. As it was already shown in the case of YAG nanocrystals, due to the spatial segregation of different vanadium oxidation states within the nanoparticle, the emission intensity ratio of V^5+^ to V^3+^ can be easily modulated through size of the nanoparticles. Moreover, the involvement of CA during the synthesis caused the reduction of oxidation state of vanadium substrate, namely from pentavalent to trivalent valence states, being observed by the changes of color of the solution, from yellow to blue, respectively (Kniec and Marciniak, 2018a,b). Citric acid is a complexing agent, which is highly soluble in polar solvents and widely used in the synthesis of nanoparticles, guaranteeing the incorporation of each metal into the material, maintaining local stoichiometry (Davar et al., 2013), which is possible due the presence of three carboxylic group (−COOH) and one hydroxyl group (−OH) in the CA chain (Gutierrez et al., 2015; Kniec and Marciniak, 2018a,b). It is well-known as a reducing and capping agent, providing possibility of charge modulation, nano-sized particles and relatively good size distribution, due the high chemical stability, steric impediment and generation of electrostatic repulsive forces (Zhang and Gao, 2004; Thio et al., 2011; Davar et al., 2013; Gutierrez et al., 2015; Shinohara et al., 2018). However, the addition of PEG, entering into polyesterification with CA, leads to discoloration of the solution (Kniec and Marciniak, 2018a,b). This phenomenon may be explained as a decrease of reducing properties of CA, resulting from the interaction between carboxylic and hydroxyl group of CA and PEG, respectively. Another approach which was frequently used in terms of stabilization of oxidation state of vanadium ions in the crystals but never in the case of nanocrystals is charge compensation. In this paper the potential possibilities of the modulation of vanadium oxidation states by varying charge compensation process, annealing temperature and the choice of appropriate synthesis conditions have been presented. Employment of these modifications leads to the changes in vanadium valence states and in consequence modify spectroscopic properties, such as emission color.

## Experimental

### LaGaO_3_:V Synthesis With Different Amounts of Citric Acid

The LaGaO_3_:xV nanocrystals have been successfully synthesized by the use of citric acid assisted sol-gel method. The first steps of preparation were carried out analogously to the previous synthesis of LaGaO_3_ (see Table S1) (Kniec and Marciniak, 2018a). Lanthanum oxide (La_2_O_3_ with 99.999% purity from Stanford Materials Corporation), gallium nitrate nonahydrate [Ga(NO_3_)_3_·9H_2_O with 99.999% purity from Alfa Aesar], ammonium metavanadate (NH_4_VO_3_ with 99% purity from Alfa Aesar) and citric acid (CA, C_6_H_8_O_7_, 99.5+% purity from Alfa Aesar,) were used as substrates of the reaction. Stoichiometric amount of lanthanum oxide was dissolved in distilled water and ultrapure nitric acid (96%). The created lanthanum nitrate was recrystallized three times using small quantities of distilled water. After this, adequate quantities of Ga(NO_3_)_3_·9H_2_O and NH_4_VO_3_ were diluted in distilled water and added to aqueous solution of obtained lanthanum nitrate. The mixture was stirred with citric acid using magnetic stirrer and heated up to 90°C for 1 h to provide the metal complexation process. Afterwards, the solution was dried for 1 week at 90°C to create a resin. Finally, the nanocrystals were received by annealing the resin in air for 8 h at 800, 900, 1,000, and 1,100°C, respectively. The vanadium dopant was used in the amount of x = 0.1% in respect to number of moles of Ga^3+^ ions. The CA were used in different molar ratio in respect to all metals (M), namely 1:1, 2:1, 4:1, 6:1, 8:1, and 10:1.

### LaGaO_3_:V Synthesis With the Charge Compensation Using Mg^2+^ and Ca^2+^ions

The LaGaO_3_:V nanocrystals with Mg^2+^ and Ca^2+^ ions as a charge compensators have been successfully obtained using the same Pechini method, which was exploited to synthesized the previous presented LaGaO_3_:V powders (Kniec and Marciniak, 2018a). The lanthanum oxide was recrystallized. Appropriate quantities of Ga(NO_3_)_3_·9H_2_O, NH_4_VO_3_, magnesium nitrate heksahydrate [Mg(NO_3_)_2_·6H_2_O with 99.999% purity from Alfa Aesar] or calcium nitrate tetrahydrate [Ca(NO_3_)_2_·4H_2_O with 99.995% purity from Alfa Aesar] were added to the mixture of all reactants and stirred with citric acid for 1 h at 90°C, where the citric acid was used in the 6-fold excess in respect to total amount of metals moles. Than appropriate volume of PEG-200 (1 PEG-200: 1 CA) was dropped to the solution. The reaction was carried out for 2 h with simultaneous heating. After this synthesis the received solutions were dried for 1 week at 90°C. The powders of LaGaO_3_:V, Mg^2+^(Ca^2+^) were finally obtained by annealing in air for 8 h at 800°C. The concentration of V ions was 0.1% in respect to number of moles of Ga^2+^ ions, whereas the total amount of Mg^2+^ (Ca^2+^) was used in the ratio of 1:1, 2:1, 4:1, and 8:1 in respect to the vanadium ions.

### Characterization

Powder diffraction studies were carried out on PANalytical X'Pert Pro diffractometer equipped with Anton Paar TCU 1000 N Temperature Control Unit using Ni-filtered Cu *K*α radiation (*V* = 40 kV, *I* = 30 mA).

Transmission electron microscopy images were obtained using the Tecnai G2 20 S/TEM Microscope from FEI Company. The microscope was equipped with a thermionic LaB6 emitter and EDS detector for elemental analysis. The study was conducted in the TEM mode at maximum voltage of 200 kV. Micrographs were taken at various magnifications, including high resolution images with lattice fringes.

The emission spectra were measured using the 266 nm excitation line from a laser diode (LD) and a Silver-Nova Super Range TEC Spectrometer form Stellarnet (1 nm spectral resolution) as a detector.

## Results and Discussion

**Figure 1:**
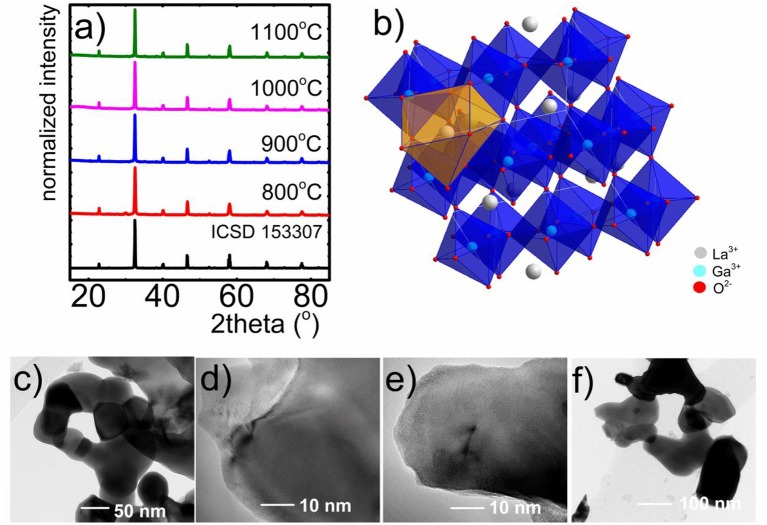
XRD patterns of LaGaO_3_:V nanocrystals synthesized by citric acid-assisted sol-gel method (M:CA 1:1) annealed at different temperatures **(a)**; visualization of LaGaO_3_ perovskite structure **(b)**; respective TEM images of LaGaO_3_:V nanocrystals with the 10-fold excess of citric acid, annealed at 800°C **(c)**, 900°C **(d)**, 1,000°C **(e)**, and 1,100°C **(f)**.

The phase purity and crystalline structure of obtained LaGaO_3_:V powders with different amounts of CA and the employment of charge compensating ions was examined using the XRD analysis (Figure 1a, see also Figures S1, S2). Moreover, the influence of annealing temperature on the LaGaO_3_ structure, especially on the grain size, was analyzed (Figures 1a,c-f). Comparing the reference peaks (ICSD 153307) with measured XRD patterns it was confirmed that obtained phosphors crystalized in orthorhombic structure and centrosymmetric Pbnm space group. Additional reflection peaks found for the sample annealed at 800°C, without charge compensation, originate from La_2_O_3_ and Ga_2_O_3_ impurities. These results confirm that the sol-gel citric acid-assisted synthesis with and without charge compensation allows to obtain the LaGaO_3_:V nanocrystals of high structural purity. The analyzed structure consists of 6-fold coordinated Ga^3+^ (GaO_6_)^9−^ and 8-fold coordinated La^3+^ ions, where La^3+^ sites are situated between slightly tilted and distorted (GaO_6_)^9−^ layers (Marti et al., 1994; Ryba-Romanowski et al., 1999a,b; Kamal et al., 2017; Kniec and Marciniak, 2018a) (Figure 1b). As it was mentioned in the previous work (Kniec and Marciniak, 2018a), due the similarities in valence states and ionic radii between Ga^3+^ and V ions (0.76, 0.78, 0.72, and 0.54 Å Kniec and Marciniak, 2018a, for Ga^3+^, V^3+^, V^4+^, and V^5+^, respectively) V occupy octahedral site of Ga^3+^ ions in the LaGaO_3_ matrix. Representative TEM images of the nanocrystals (Figures 1c–f), which were synthesized using the citric acid-assisted sol-gel method reveal well-crystalized agglomerated nanoparticles. Additionally it can be noticed that this synthesis method provides higher degree of grains dispersion and their separation in respect to the previously described modified Pechini method (Kniec and Marciniak, 2018a). According to our predictions the increase of annealing temperature results in the enlargement of the average grain size: from 66 nm for 800°C, 79 for 900°C, 114 nm for 1,000°C to 145 nm for 1,100°C. It is worth mentioning, that the highest grain size of LaGaO_3_:V annealed at 1,100°C is more than 2-fold smaller in respect to the counterpart synthesized using Pechini method (381.8 nm) (Kniec and Marciniak, 2018a). However, the increase of CA does not cause the evident changes in grain size.

The first approach employed by us to control the valence state of vanadium ions was compensation method. Two series of nanocrystals with different molar ratio of Mg^2+^ and Ca^2+^ ions in respect to the V ions were prepared. Proposed charge compensation can be written as follows:

(1) $$\begin{array}{r} 2\times Ga^{3+}\to 1\times V^{4+}+1\times Ca^{2+}/Mg^{2+} \end{array}$$

**Figure 2:**
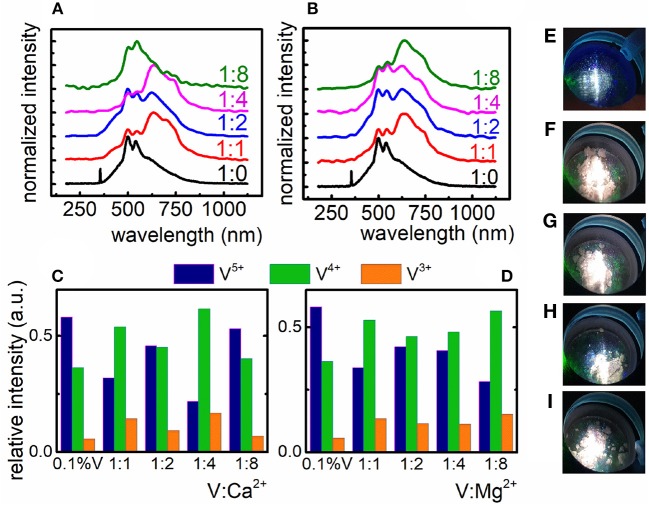
Influence of the Ca^2+^ **(A)** and Mg^2+^ **(B)** compensating ions on the V emission spectrum in LaGaO_3_ nanocrystals; relative emission intensity of the V ions after charge compensation with Ca^2+^ **(C)** and Mg^2+^ ions **(D)**; emission color of uncompensated LaGaO_3_:V nanocrystals **(E)** and compensated LaGaO_3_:V nanocrystals with the V:Mg^2+^ ratio of 1:1, 1:2, 1:4, and 1:8 **(F–I)**, respectively.

Two Ga^3+^ sites were substituted by one V^4+^ ion and one Ca^2+^/Mg^2+^ ion so that the excess electron could be transferred to the compensating ion. Taking into consideration the structural properties of compensated material, it is worth mentioning, that the introduction of even large excess of Ca^2+^/Mg^2+^ ions does not influence the changes of XRD patterns (Figure S1). According to our predictions, the introduction of Mg^2+^ and Ca^2+^ in the crystal structure caused the rise of the V^4+^ emission intensity (λ_em_ = 633 nm) in respect to the uncompensated counterpart (Figures 2A–D). Due to the fact that no structural changes can be found in the XRD pattern even for high Ca^2+^/Mg^2+^, the observed changes are the confirmation of the successful charge compensation and thus the increase of V^4+^ concentration. One can notice that even a small addition of Mg^2+^ and Ca^2+^ ions to the LaGaO_3_:0.1%V crystal lattice affects the shape of emission spectra (Figures 2C,D) significantly. It can be found that Mg^2+^ ions revealed better, in respect to the Ca^2+^ ions, performance to charge compensation which is reflected in the dominant emission of V^4+^ ions over the emission of V^3+^ and V^5+^ ions for each amount of compensating ion. However, the most satisfactory charge compensation was found in the case of incorporation of 8-fold excess of Mg^2+^ in respect to V (Figures 2B,D). This effect may be explained in terms of superior lability of Mg^2+^ ions in the lattice, which results from their smaller ionic radius (0.78 Å) compared to Ca^2+^ ions (1.06 Å). The impact of the amount of compensating ions on the emission intensity of the particular oxidation state of vanadium ions is presented in Figures 2C,D. In the case of Mg^2+^ gradual increase of its concentration causes gradual decrease of V^5+^ emission intensity and enhancement of V^4+^ intensity while V^3+^ becomes almost constant. The changes observed in the case of Ca^2+^ are rather irregular. The consequence of observed charge compensation is the modulation of emission color (Figures 2E–I). It is also worth noting that in the case of calcium ions there is a concentration limit (1:8), above which V^5+^ emission intensity becomes anew dominant (Figures 2A,C).

The incorporation of compensating ions caused the enhancement of V^4+^ emission intensity, however the luminescence of both V^5+^ and V^3+^ ions is still observed and cannot be completely reduced. The contribution of V^5+^ emission intensity in the emission spectra of LaGaO_3_:V is relevant and that is why the emission color does not changes significantly (Figures 2E–I) indicating that charge compensation does not provide sufficient ability to modulation of vanadium oxidation states. Based on these results new approach to charge modulation was proposed.

**Figure 3:**
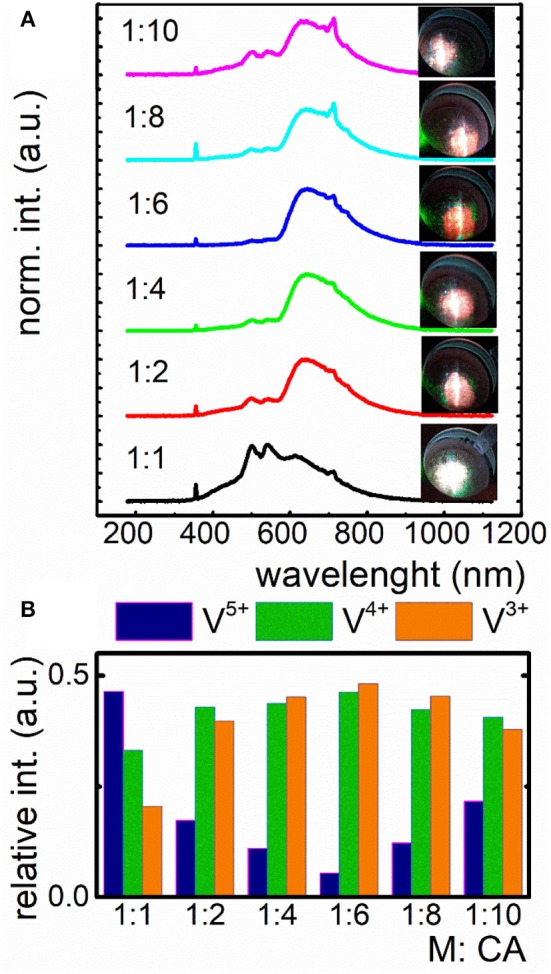
Influence of the different amount of citric acid (CA) in respect to V ions on the emission spectrum of nanocrystalline LaGaO_3_:V and the corresponding emission color **(A)**; contribution of emission intensity of particular V ions in the total luminescence of LaGaO_3_:V nanocrystals **(B)**.

Taking advantage from the fact that PEG, which was used in the case of previously described Pechini method is well-known from its oxidizing properties and may lead to the increase of V^5+^ concentration, in the second approach we decided to use sol-gel method to eliminate PEG as a reagent, being involved in the resin creation. The issue was to involve all COOH groups to reduction reaction. Cit^3−^ ions were used in different molar quantity in respect to the total amount of metal ions (M) in the lattice (Figure 3). To verify the capability of V oxidation states modulation by the CA concentration, the emission spectra of LaGaO_3_:V nanocrystals were recorded at −150°C under 266 nm excitation. Low measurement temperature provides the highest emission intensity, being to a lesser extent affected by lattice vibrations, reducing the luminescence temperature quenching (Kniec and Marciniak, 2018a,b). The employment of the same molar amounts of ions and CA caused the presence of three emitting V oxidation states, namely V^5+^, V^4+^, and V^3+^, with the predominant emission intensity of V^5+^. This phenomenon indicates that this molar ratio is insufficient to provide significant reduction of V^5+^. The employment of 2-fold excess of CA (1:2) leads to the apparent domination of V^4+^ emission and thereby the change of emission color (Figures 3A,B), pointing to the immense impact on the V luminescence properties. Increasing the quantity of capping agent to 6-fold excess in respect to metal ions, the V^5+^ emission intensity decreases with the simultaneously rise of V^4+^ and V^3+^ luminescence (Figure 3B), being a limit value, above which a reversed dependence occurs. This phenomenon determines the color output of LaGaO_3_:V, which changes from white to red, while the emission is being red-shifted (V^4+^ and V^3+^) and becomes whitish as the V^5+^ amount increases (Figure 3A, see also Figures S3, S5). It is worth noting that although initially with the increase of CA concentration the emission intensity of V^5+^ decreases in respect to the V^4+^ and V^3+^, above 1:8 ratio the V^5+^ emission band appears anew. The enhancement of V^5+^ luminescence may be due the fact, that higher content of Cit^3−^ provides the stabilization of more positive charge on V. In turn the V charge lowering confirms the reducing properties of CA even in the nanoscale materials. On the other hand, as it was already proved in the case of nanocrystalline phosphors doped with vanadium ions the V^5+^ ions are mainly located in the surface part of the grains, while V^4+^ and V^3+^ in the core part (Kniec and Marciniak, 2018b). Therefore, the enhancement of the V^5+^ emission at higher CA concentration may be related with the higher dispersion of the LaGaO_3_ nanocrystals leading also to the higher separation of the grains. This is in agreement with the TEM images presented in Figure S4. The observed changes of the relative emission intensity of particular V oxidation state causes modulation of the emission color (Figures 3A,B).

**Figure 4:**
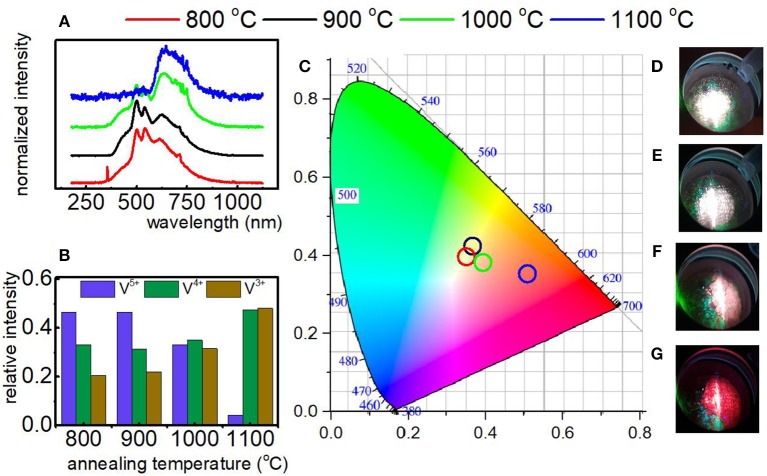
Emission spectrum of LaGaO_3_:V nanocrystals as a function of annealing temperature recorded at −150°C under 266 nm excitation **(A)**; the relative emission of each V ions in the LaGaO_3_ nanocrystals annealed at different temperatures **(B)**; the CIE 1,931 chromatic coordinates calculated for LaGaO_3_:V nanocrystals annealed at different temperatures **(C)**; LaGaO_3_:V emission colors at −150°C after annealing at 800, 900, 1,000, and 1,100°C **(D–G)**, respectively.

In the course of our studies it was found that in the case of YAG:V and LaGaO_3_:V nanocrystals the vanadium on its higher oxidation state is mainly located at the surface part of the grain. Therefore, in the last approach we verified either size of the nanocrystals may enable the reduction of the V^5+^ emission intensity in respect to the V^4+^ and V^3+^. The size of the nanocrystals was controlled by the annealing temperature for constant molar ratio of CA:V (1:1). As it can be observed the increase of grain size causes gradual increase of bands associated with the ^2^E → ^2^T_2_ and ^1^E_2_ → ^3^T_1g_ electronic transitions of V^4+^ and V^3+^, respectively in respect to the V^4+^ → O^2−^ band of V^5+^ (Figure 4A). This is due to the fact that the rise of grain size causes a decrease in the surface-to-volume ratio of the nanocrystals and thus a drop of the amount of emitting V^5+^ ions compared to V^4+^ and V^3+^ ions. The most apparent emission changes take part by the annealing temperature increase from 1,000 to 1,100°C, whereas least relevant are between 800 and 900°C (Figure 4B), being strictly related to enlargement of the grain size by 42.94 and 2.53 nm, respectively. Therefore, the emission color changes can be observed, ranging from white, connected with the domination of V^5+^ luminescence, trough pinkish, related to the presence of each V ions, ending on red color, originating from predominant V^4+^ and V^3+^ luminescence (Figures 4B,D–G). This in turn enables the modulation of color output of LaGaO_3_:V, which varies depending on the total contribution of each V ions in the luminescence (Figures 4B,D–G). The contribution of each V^n+^ ions into total emission spectra was estimated by the calculation of the integrals in the range of 400–570, 580–675, and 680–750 nm integral emission intensity for V^3+^, V^4+^, and V^5+^, respectively. In addition, knowing the average grain size of LaGaO_3_:V nanocrystals, the possibility of establishment of the emission spectrum and, consequently, predict the color output was affirmed (Figure 4C). The same dependence, concerning the size effect on the V oxidation states was also observed for LaGaO_3_:V nanocrystals, where citric acid was incorporated in higher concentration (see Figure S3).

As it can be observed, that all presented approaches led to the decrease of V^5+^ emission intensity, however the last method, based on the modification of the sizes of the LaGaO_3_ nanocrystals, is the most efficient.

## Conclusion

In this paper three approaches to stabilize and modulate the V oxidation states in LaGaO_3_ nanocrystals and thus their emission color have been demonstrated. It was found that in this perovskite lattice three different V valence states are present, namely V^5+^, V^4+^, and V^3+^, showing the emission, being related to the V^4+^ → O^2−^ CT transition (λ_em_ = 520 nm), ^2^E → ^2^T_2_ (λ_em_ = 645 nm) and narrow line ^1^E_2_ → ^3^T_1g_ (λ_em_ = 712 nm) d-d electronic transition, respectively.

Furthermore it was concluded that at −150°C under 266 irradiation V^4+^ ions exhibit broad band emission, whereas V^3+^ ions reveals narrow line luminescence. It was presented that three different approaches including the implementation of compensating ions, by altering the ratio of citric acid to metal ions and the tuning of the size of the nanocrystals in range 66–145 nm through change of annealing temperature in 800–1,100°C range may provide the ability to regulate the valence state of vanadium ions. It was found that in the case of charge compensation method the introducing of Mg^2+^ ions is much more efficient, due the similar ionic radius to V ions and higher lability of compensating ions in respect to Ca^2+^. In turn the citric acid-assisted synthesis, where CA was used in the excess in respect to total amount of metals in the lattice, leads to the significant increase of V^4+^ and V^3+^ luminescent intensity with the simultaneous improvement of nanocrystals separation and narrower size distribution in respect to the powders obtained using Pechini method. Basing on the fact, that particular oxidation states of V ions are localized in different part of the LaGaO_3_ nanocrystals, namely V^5+^ in the surface, V^4+^ and V^3+^ in the core part, respectively, the influence of the grain size of V emission intensity was investigated. The increase of the annealing temperature and thereby the size of the nanocrystals, leads to the decrease of V^5+^ luminescence and simultaneously causing the enhancement of V^4+^ and V^3+^ ones. The most significant V^5+^ emission changes are observed when the annealing temperature increase from 1,000 to 1,100°C, which corresponds to the enlargement of the grain size by 43 nm. All of the presented approaches provide the differences of V emission intensity, however the most efficient method based on the modification of the grain size, which is confirmed by the most apparent changes in LaGaO_3_:V color output. Taking into account that different oxidation states of vanadium ions possess favorable optical properties for i.e., lightning and luminescent thermometry, we believe that this study may be of relevant importance for further application of vanadium based nanocrystalline phosphors.

## Data Availability

All datasets generated for this study are included in the manuscript and the Supplementary Files.

## Author Contributions

All authors listed have made a substantial, direct and intellectual contribution to the work, and approved it for publication.

### Conflict of Interest Statement

The authors declare that the research was conducted in the absence of any commercial or financial relationships that could be construed as a potential conflict of interest.
