# Supplementary material for: Different Strategies of Stabilization of Vanadium Oxidation States in Lagao3 Nanocrystals
Source: Front Chem. 2019 Jul 23;7:520. doi: 10.3389/fchem.2019.00520 (PMC6664484; doi:10.3389/fchem.2019.00520)
Supplement: Supplementary file 1 [file Table_1.docx]

**Supporting information**

**Different strategies of stabilization of vanadium oxidation states in LaGaO_3_ nanocrystals**

**K.Kniec^1*^, L. Marciniak^1*^**

^1^Institute of Low Temperature and Structure Research, Polish Academy of Sciences, Okólna 2, 50-422 Wroclaw, Poland

* corresponding author: *k.kniec@intibs.pl*, *l.marciniak@intibs.pl*

KEYWORDS vanadium, charge compensation, citric acid, luminescence, nanocrystals

Table. S1. Quantitates of all substrates taken to the synthesis.

| **Charge compensation method** | | | | | | | | | | |
| --- | --- | --- | --- | --- | --- | --- | --- | --- | --- | --- |
| V: Mg | La_2_O_3_ [g] | Ga(NO_3_)_3_^.^ 9H_2_O [g] | NH_4_VO_3_ [g] | Mg(NO_3_)_2_^.^ 6H_2_O [g] | CA [g] | | PEG-200 [g] | Annealing temperature | | Time of annealing process |
| 1:1 | 0.6348 | 1.627 | 0.0005 | 0.001 | 1.1 | | 1.2 | 800^o^C | | 8 h |
| 1:2 | 0.6348 | 1.627 | 0.0005 | 0.002 | 1.1 | | 1.2 | 800^o^C | | 8 h |
| 1:4 | 0.6348 | 1.627 | 0.0005 | 0.004 | 1.1 | | 1.2 | 800^o^C | | 8 h |
| 1:8 | 0.6348 | 1.627 | 0.0005 | 0.008 | 1.1 | | 1.2 | 800^o^C | | 8 h |
| V: Ca | La_2_O_3_ [g] | Ga(NO_3_)_3_^.^ 9H_2_O [g] | NH_4_VO_3_ [g] | Ca(NO_3_)_2_^.^ 4H_2_O [g] | CA [g] | | PEG-200 [g] | Annealing temperature | | Time of annealing process |
| 1:1 | 0.6348 | 1.627 | 0.0005 | 0.0009 | 1.1 | | 1.2 | 800^o^C | | 8 h |
| 1:2 | 0.6348 | 1.627 | 0.0005 | 0.0018 | 1.1 | | 1.2 | 800^o^C | | 8 h |
| 1:4 | 0.6348 | 1.627 | 0.0005 | 0.0036 | 1.1 | | 1.2 | 800^o^C | | 8 h |
| 1:8 | 0.6348 | 1.627 | 0.0005 | 0.0072 | 1.1 | | 1.2 | 800^o^C | | 8 h |
| **CA-assisted synthesis** | | | | | | | | | | |
| M: CA | La_2_O_3_ [g] | Ga(NO_3_)_3_^.^ 9H_2_O [g] | NH_4_VO_3_ [g] | CA [g] | | Annealing temperature | | | Time of annealing process | |
| 1:1 | 0.6348 | 1.627 | 0.0005 | 1.1 | | 800^o^C | | | 8 h | |
| 1:2 | 0.6348 | 1.627 | 0.0005 | 2.2 | | 800^o^C | | | 8 h | |
| 1:4 | 0.6348 | 1.627 | 0.0005 | 4.4 | | 800^o^C | | | 8 h | |
| 1:6 | 0.6348 | 1.627 | 0.0005 | 6.7 | | 800^o^C | | | 8 h | |
| 1:8 | 0.6348 | 1.627 | 0.0005 | 9.0 | | 800^o^C | | | 8 h | |
| 1:10 | 0.6348 | 1.627 | 0.0005 | 11.2 | | 800^o^C | | | 8 h | |
| **Grain size tuning** | | | | | | | | | | |
| M: CA | La_2_O_3_ [g] | Ga(NO_3_)_3_^.^ 9H_2_O [g] | NH_4_VO_3_ [g] | CA [g] | | Annealing temperature | | | Time of annealing process | |
| 1:1 | 0.6348 | 1.627 | 0.0005 | 1.1 | | 800^o^C, 900^o^C, 1000^o^C, 1100^o^C | | | 8 h | |
| 1:2 | 0.6348 | 1.627 | 0.0005 | 2.2 | | 800^o^C, 900^o^C, 1000^o^C, 1100^o^C | | | 8 h | |
| 1:4 | 0.6348 | 1.627 | 0.0005 | 4.4 | | 800^o^C, 900^o^C, 1000^o^C, 1100^o^C | | | 8 h | |
| 1:6 | 0.6348 | 1.627 | 0.0005 | 6.7 | | 800^o^C, 900^o^C, 1000^o^C, 1100^o^C | | | 8 h | |
| 1:8 | 0.6348 | 1.627 | 0.0005 | 9.0 | | 800^o^C, 900^o^C, 1000^o^C, 1100^o^C | | | 8 h | |
| 1:10 | 0.6348 | 1.627 | 0.0005 | 11.2 | | 800^o^C, 900^o^C, 1000^o^C, 1100^o^C | | | 8 h | |





Fig. S1. XRD patterns of LaGaO_3_:0.1% V nanocrystals with different molar ratio of V ions in respect to Mg^2+^/Ca^2+^ ions.





Fig. S2. XRD patterns of LaGaO_3_:0.1% nanocrystals annealed at 800^o^C- a), 900^o^C -b), 1000^o^C -c) and 1000^o^C- d), respectively.





Fig. S3. Emission spectra of LaGaO_3_:0.1% nanocrystals synthesized with different amount of CA, recorded at -150^o^C.


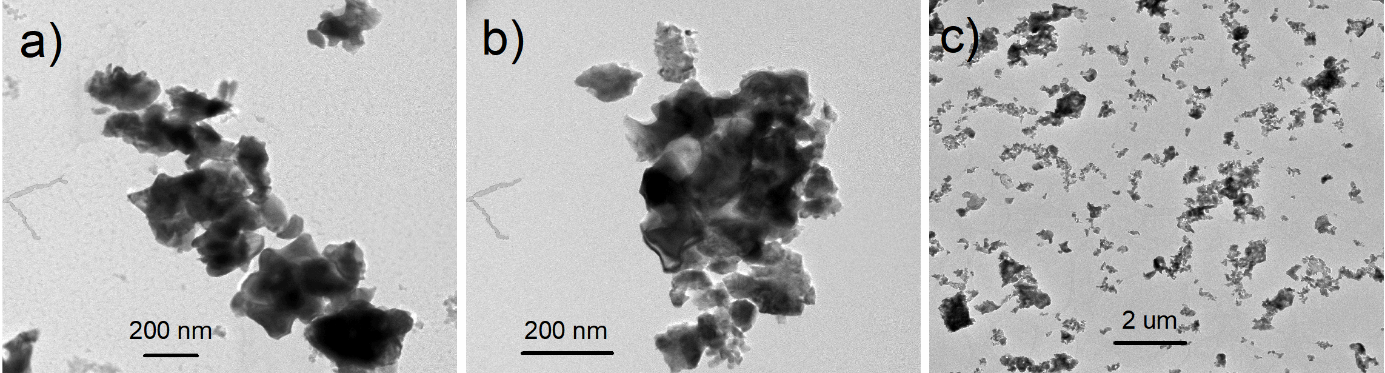


Fig. S4. TEM images of LaGaO_3_:0.1%V nanocrystals annealed at 800^o^C with different excess of CA in respect to total amount of metals, namely 1:1 –a), 6:1 –b) and 10:1 -c).





Fig. S5. Relative emission intensity of metal ions in LaGaO_3_ nanocrystals in different molar ratio in respect to CA.
